# High accumulation of γ-linolenic acid and Stearidonic acid in transgenic Perilla (*Perilla frutescens var. frutescens*) seeds

**DOI:** 10.1186/s12870-019-1713-2

**Published:** 2019-04-01

**Authors:** Kyeong-Ryeol Lee, Kyung-Hwan Kim, Jung Bong Kim, Seung-Bum Hong, Inhwa Jeon, Hyun Uk Kim, Myung Hee Lee, Jae Kwang Kim

**Affiliations:** 1 Department of Agricultural Biotechnology, National Institute of Agricultural Science, RDA, Jeonju, 54874 Jeollabukdo Republic of Korea; 2 Department of Agro-food Resources, National Institute of Agricultural Science, RDA, Wanju-gun, Jeollabukdo Republic of Korea; 3 Department of Agricultural Biology, National Institute of Agricultural Science, RDA, Wanju-gun, Jeollabukdo Republic of Korea; 4 Department of Bioindustry and Bioresource Engineering, Plant Engineering Research Institute, Sejong University, Seoul, 05006 Republic of Korea; 5 Department of Southern Area Crop Science, National Institute of Crop Science, Miryang, Gyeongsangnamdo Republic of Korea; 6 Division of Life Sciences, Incheon National University, Incheon, Republic of Korea

**Keywords:** Delta 6 desaturase, γ-Linolenic acid, Stearidonic acid, Perilla, *Phytophthora citrophthora*

## Abstract

**Background:**

Polyunsaturated fatty acids such as linoleic acid (LA) and α-linolenic acid (ALA) are abundant in vegetable oils and are important for human health. In the body, LA and ALA are respectively converted to the omega-6 fatty acid γ-linolenic acid (GLA) and the omega-3 fatty acid stearidonic acid (SDA) by Δ6 desaturase (D6DES). Currently, dietary GLA and SDA are mainly obtained from marine organisms, but given their benefits to human health, many studies have aimed to enhance their accumulation in transgenic crops. *Perilla frutescens* (perilla) accumulates more ALA in its seed oil compared to other oilseed crops, making it a good candidate for the production of fatty acids via the fatty acid desaturase D6DES.

**Results:**

In this study, we cloned the *D6DES* gene from *Phytophthora citrophthora* and confirmed its function in budding yeast. We then transformed the functional *D6DES* gene under the control of the seed-specific *vicilin* promoter into the perilla cultivar Yeobsil. The resulting transgenic perilla seeds accumulated significant levels of GLA and SDA, as well as putative C18:2Δ^6,9^ at minor levels. Developing seeds and leaves also accumulated GLA and SDA, although *PcD6DES* expression and GLA and SDA levels were much lower in leaves compared to developing seeds. GLA and SDA accumulated in both polar lipids and neutral lipids in mature perilla seeds expressing *PcD6DES*, especially in neutral lipids. Although the seed weight in *PcD6DES* perilla was 87–96% that of wild type, the total oil content per seed weight was similar between lines. The *PcD6DES* perilla plants contained very high content (over 45%) of both GLA and SDA in seed oil.

**Conclusions:**

Thus, *PcD6DES* perilla plants may represent a feasible alternative to traditional marine sources for the production of omega-3 oil capsules and to evening primrose seed oil for GLA as health food. In addition, these plants can be used to create other transgenic lines harboring additional genes to produce other desirable fish-oil like oils.

**Electronic supplementary material:**

The online version of this article (10.1186/s12870-019-1713-2) contains supplementary material, which is available to authorized users.

## Background

Along with high proportions of other fatty acids from plants, polyunsaturated fatty acids (PUFAs) such as cis-9,12-octadecadienoic acid (linoleic acid; LA) and cis-9,12,15-octadecatrienoic acid (α-linolenic acid; ALA) are essential to human health. These PUFAs are used to produce cis-6,9,12-octadecatrienoic acid (γ-linolenic acid; GLA) and cis-6,9,12,15-octadecatrienoic acid (stearidonic acid; SDA), which are health-promoting molecules as well as serving as precursors for the very long chain polyunsaturated fatty acids (VLCPUFAs) found in algae, fungi and vertebrates, including fish and human. GLA is an omega-6 fatty acid that acts as an anti-inflammatory agent and relieves skin problems, such as atopy [1, 2]. SDA is an omega-3 fatty acid that serves a precursor for other omega-3 fatty acids such as cis-5,8,11,14,17-eicosapentaenoic acid (EPA) and cis-4,7,10,13,16,19-docosahexaenoic acid (DHA), a representative ω-3 VLCPUFAs. In humans, dietary SDA is more efficiently converted to EPA compared to ALA [3], and SDA therefore provides similar health benefits to humans as ω-3 VLCPUFAs [4]. In addition, SDA has been characterized as a potent inhibitor of cancer cell growth and an effective molecule against skin inflammation and atopic dermatitis that also prevents hypertriglyceridemia [5].

As humans can synthesize DHA from either ALA or SDA, it may be beneficial for humans to take in high levels of ALA and/or SDA through their diet. GLA and/or SDA are present in the seed oils of several plant species, such as evening primrose (*Oenothera biennis*) [6], blackcurrant (*Ribes nigrum*) [7], *Primula* spp. [8], *Echium* spp. [9], hemp (*Cannabis sativa*) [10] and Boraginaceae plants [11], including borage (*Borago officinalis*). However, these wild plants do not produce large amounts of oil, and they produce only low levels of GLA and SDA. Consequentially, GLA- and SDA-containing health foods are rather expensive.

GLA and SDA are converted from LA and ALA, respectively, through desaturation at the sixth carbon, a process catalyzed by Δ6 desaturase (D6DES). Fungal D6DES can also convert oleic acid to C18:2Δ^6,9^ [12, 13], and ALA-specific D6DES from *Primula* spp. synthesizes only SDA [14, 15]. The *D6DES* gene, which was first identified in a cyanobacterium [16], encodes a divergent form of the sphingolipid Δ8-desaturase [17]. Subsequently, *D6DES* genes have been identified in bacteria, algae, fungi, mosses, vertebrates and several plant species [18]. Fatty acid desaturases involved in the synthesis of VLCPUFA, including D6DES, are “front-end” desaturases that contain a cytochrome *b5* (cyb5) domain at their N-termini, which is essential for fatty acid desaturation [19–21].

Perilla (*Perilla frutescens var. frutescens*) is an annual herbaceous plant belonging to the mint family, Lamiaceae. Perilla has been widely cultivated as an oilseed crop and leaf vegetable in East Asia. Perilla seed oil is used as both an edible and an industrial crop in products such as paint, varnish and ink [22]. Perilla seeds comprise 35–45% oil and accumulate one of the highest proportions of ALA (54–64%) in the plant kingdom [22]. The mechanism underlying the production of high levels of ALA is of interest, and thus, transcriptome analysis has been performed to identify perilla genes involved in ALA bisoynthesis and accumulation [23]. PUFAs represent approximately 80% of the total fatty acid composition in perilla seeds, which could confer health benefits for humans [24]. We therefore hypothesized that if perilla were transformed with the *D6DES* gene, it would produce high amounts of GLA and/or SDA and serve as a feasible resource for large-scale GLA and/or SDA production. Furthermore, the seeds from these transgenic crops could potentially be used for effective, large-scale production of EPA or DHA.

In this study, we confirmed that D6DES from the fungus *Phytophthora citrophthora* synthesizes GLA and SDA from LA and ALA, respectively, in budding yeast. We further revealed that transgenic perilla seeds expressing *PcD6DES* produced very high levels of GLA and SDA, with each accounting for over 20% of the seed oil content. Our results indicate that *PcD6DES* and perilla are an effective gene and host combination that could be utilized to efficiently produce GLA and SDA.

## Methods

### Plant material

*Perilla frutescens var. frutescens* cv. Yeobsil was grown in a greenhouse and the seeds were harvested for transformation. The seeds were surface sterilized in 70% (*v*/v) ethanol for 1 min, followed by 4-fold diluted commercial bleach for 20 min, washed three times with sterile distilled water and immersed in sterile distilled water for 2 h at room temperature. After brief drying on sterile filter paper, the seeds were placed on Murashige and Skoog (MS) basal medium [25] supplemented with 30 g/L sucrose and 0.4% (*w*/*v*) Phytagel (Sigma, USA) with the pH adjusted to 5.8 before autoclaving. Surface sterilized seeds were germinated in a culture room at 28 °C under a 16 h:8 h (light: dark) photoperiod.

### Gene cloning

The *D6DES* gene was cloned from *P. citrophthora* (KACC 40188), which was obtained from the National Agrobiodiversity Center, National Institute of Agricultural Science, RDA, Republic of Korea. Total RNA was extracted from *P. citrophthora* for *D6DES* gene cloning. First-strand cDNA was synthesized from total RNA using a PrimeScript™ 1st strand cDNA Synthesis Kit (Takara, Japan) following the manufacturer’s protocol. Degenerate PCR and 5′−/3′-RACE PCR were used for cloning. Primer sequences for degenerate PCR were designed based on consensus sequences of *D6DES* from the fungus *Mucor circinelloides* and *Rhizopus stolonifera* var. *stolonifer*. Degenerate PCR was performed at 94 °C for 5 min, followed by 30 cycles of 94 °C for 20 s, 54 °C for 30 s and 72 °C for 1 min, with an additional extension at 72 °C for 5 min. PCR amplification was performed with the degenerate primers using Ex Taq polymerase (Takara, Japan). RACE PCR was performed using a SMART RACE cDNA Amplification Kit (Clontech, USA) following the manufacturer’s protocol. The *D6DES* gene from *P. citrophthora* (*PcD6DES*) was registered in GenBank NCBI under Accession No. DQ836059. Primers used in these procedures are listed in Additional file 1: Table S1.

### Vector construction

To confirm the function of *PcD6DES* in budding yeast (*Saccharomyces cerevisiae*), the *PcD6DES* gene was subcloned in between the *Hin*dIII and *Bam*HI sites of the pYES2/CT vector (Invitrogen, USA) harboring the galactose-inducible *GAL1* promoter and *URA3* gene. The complete vector was named pYES2-*PcD6DES*. The process used to transform and express the *PcD6DES* gene in perilla was as follows: the pVicOCS vector containing the seed-specific *vicilin* promoter and *octopine synthase III* (*OCSIII*) terminator was digested with *Bam*HI and *Hin*dIII, and the ORF of *PcD6DES* was inserted between the *vicilin* promoter and *OCSIII* terminator. The vicilin promoter-*PcD6DES*-OCSIII terminator cassette was inserted into the multiple cloning site of pCAMBIA3300. The complete vector was named pCAMBIA-*PcD6DES*. The destination vectors used in this study are shown in Additional file 2: Figure S1.

### Phylogenetic and transmembrane domain analysis

The sequences of *D6DES* genes registered in GenBank were collected by carrying out BLASTN with the *PcD6DES* gene sequence as a query. The phylogenetic relationship of these *D6DES* genes was analyzed using DNASTAR MegAlign (Ver. 7.2.1) via the ClustalW method. A phylogenetic tree was constructed from the alignment data using TreeView (Ver. 1.6.6). The transmembrane domain was predicted with TOPCONS (http://topcons.cbr.su.se/).

### Yeast transformation and culture

*Saccharomyces cerevisiae* INVSc1 (Invitrogen, USA) was used for the expression of *PcD6DES* and subsequent production of GLA and SDA. Yeast transformation was carried out in accordance with the manufacturer’s protocol. Yeast culture and induction of *PcD6DES* were performed as follows: Yeast cells containing pYES2-*PcD6DES* were cultured in uracil-deficient medium containing with 2% raffinose and 1% Tergitol NP-40 at 30 °C. At O.D_600_ = 0.5–0.6, 0.5 mM of the substrates LA and ALA with 2% galactose as an inducer were added to the culture, followed incubation for 3 days at 20 °C.

### Perilla transformation

Perilla transformation was performed as described by Kim et al. [26] with slight modifications. *Agrobacterium tumefaciens* strain EHA105 harboring pCAMBIA-*PcD6DES* was inoculated in Luria Bertani broth containing 50 mg/L kanamycin and 50 mg/L rifampicin and cultured at 28 °C overnight. The cells were harvested and resuspended in liquid MS basal medium. Hypocotyl pieces 0.5 to 1 cm in length were excised from in vitro-grown seedlings and inoculated with *Agrobacterium* solution for 1 h. After brief blotting with sterile filter paper, the explants were transferred to MS basal medium supplemented with 30 g/L sucrose and 0.4% Phytagel and co-cultured for 2 days at 26 °C in the dark. After co-culture, the explants were transferred to fresh MS medium supplemented with 30 g/L sucrose, 3.0 mg/L benzyladenine, 0.01 mg/L naphthaleneacetic acid, 2 mg/L phosphinothricin, 400 mg/L carbenicillin and 0.4% Phytagel. The explants were subcultured every two weeks for 8 weeks. The shoots that regenerated on the explants were excised and transferred to fresh selective MS basal medium for shoot elongation. The elongated shoots were transferred to half-strength MS basal medium for rooting. When whole plants had formed, they were acclimated on commercial soil in an airtight container for 1 week and transferred to the greenhouse.

### Fatty acid composition analysis and the determination of seed oil content

Fatty acid extraction and FAME preparation in yeast cells were followed slightly modified version of method by Kim et al. [27]. Yeast cells were centrifuged, resuspended in 1 volume of distilled water to remove residual fatty acids (LA and ALA), harvested and lyophilized for 48 h. Lipids of freeze-dried yeast cells with 0.5 mg pentadecanoic acid were extracted with 5 mL extraction solution (chloroform: methanol = 2:1, *v*/v) via sonication at room temperature. Five milliliters of 0.58% NaCl solution was added to the mixture and centrifuged at 2000 rpm for 10 min. The supernatant was discarded and the lower phase was dried under a flow of nitrogen. Toluene (0.5 mL) and 0.5 N NaOH in methanol were added to the dried samples, and the transmethylation mixture was reacted in boiling water for 3 min. After cooling, 2 N BF_3_ in methanol was added to the mixture, which was reacted once more for 5 min. Ten milliliters of distilled water and 10 mL petroleum ether were added to the sample, followed by centrifugation. The supernatant containing fatty acid methyl esters (FAMEs) was collected for gas chromatography (GC) analysis performed on a HP 5890 (Agilent, USA) with flame ionization detector (FID) and 25 m × 0.2 mm (inner diameter) HP 20 M from 180 °C to 200 °C at 1 °C/min.

Perilla seeds were weighed and crushed with a metal stick in a glass tube. A mixture of LA (Sigma, St. Louis, MO, USA), GLA (Matreya, PA, USA), ALA (Sigma, St. Louis, MO, USA) and SDA (Santa Cruz Biotechnology, CA, USA) was used as an external standard. Seed samples and the external standard were transmethylated at 85 °C for 90 min in 0.3 mL of toluene and 1 mL of 5% H_2_SO_4_ (*v*/v) in methanol. Pentadecanoic acid (100 μg) was added to each sample as an internal standard. After transmethylation, 1.5 mL of 0.9% NaCl solution was added to the sample and the FAMEs were transferred to a new tube after extracting the sample three times with 1.5 mL of n-hexane. The FAMEs were analyzed by GC-2010 plus (Shimadzu, Japan) GC with FID and a 30 m × 0.25 mm (inner diameter) HP-FFAP column (Agilent, USA) while increasing the oven temperature from 190 °C to 230 °C at 3 °C/min. Nitrogen was used as the carrier gas in both cases for fatty acid analysis.

Seed oil contents were determined based on the results by fatty acid analyses. The formula for seed oil content is as follows. Seed oil content = (Total peak area of all seed fatty acids except internal standard)*(mass of internal standard)/(peak area of internal standard).

### GC-TOF MS analysis

Each FAME sample (1 μL) was injected into the Agilent 7890A GC with an Agilent 7683B autosampler (Agilent, Atlanta, GA, USA) with a split ratio of 25 and separated in a 30 m × 0.25 mm I.D. fused-silica capillary column coated with 0.25 μm CP-SIL 8 CB Low Bleed (Varian Inc., Palo Alto, CA, USA). The injector temperature was 230 °C. The helium gas flow rate through the column was 1.0 mL/min. The temperature program was as follows: starting temperature 80 °C, maintained for 2 min, followed by an increase to 320 °C at 15 °C/min and a 10 min hold at 320 °C. The transfer line and ion-source temperatures were 250 and 200 °C, respectively. The scanned mass range was 85–600 *m/z*, and the detector voltage was set at 1700 V.

### Thin layer chromatography (TLC)

Lipid extraction and TLC were performed based on Kim et al. [28]. Briefly, lipids were extracted with extraction solution (chloroform: methanol = 2:1, *v*/v) from ground perilla seeds and spotted onto silica gel G60 plates (Merck Millipore, USA). Lipid samples were separated using developing solvent (hexane: diethylether: acetic acid = 70:30:1, v/v/v) in a TLC developing tank for 50 min at room temperature. The silica gel plate was removed from the TLC developing tank and sprayed with 0.1% primuline (Sigma, St. Louis, MO, USA) in 80% acetone. Lipid spots were visualized on an ultraviolet transilluminator. A large spot at the top (TAG), two small spots in the middle (diacylglycerol) and two small spots around the baseline (polar lipids) were scraped off the TLC plate with a scalpel and subjected to fatty acid analysis.

### RT-PCR and quantitative RT-PCR

Total RNAs of yeast cells and perilla tissues were extracted with RNeasy Mini kit and RNeasy Plant Mini kit (Qiagen, USA), respectively. First-strand cDNA was synthesized using RNA to cDNA EcoDry Premix (Clontech, USA) following the manufacturer’s protocol. RT-PCR was carried out using ExTaq DNA polymerase (Takara, Japan) and 1 μg first-strand cDNA from yeast RNA as a template. The PCR conditions were as follows: 94 °C for 3 min, 30 cycles of 94 °C for 20 s, 55 °C for 20 s and 72 °C for 80 s, and additional extension at 72 °C for 5 min.

Quantitative RT-PCR was carried out with SYBR premix Ex Taq II (Tli RNaseH plus; Takara, Japan), and first-strand cDNA diluted 20-fold was used as template for PCR on a StepOnePlus Real-Time PCR System (Applied Biosystems, USA). The PCR conditions were as follows: 94 °C for 30 s, 40 cycles of 94 °C for 5 s, 55 °C for 20 s and 72 °C for 20 s, and an additional cycle of 94 °C for 15 s, 55 °C for 1 min, and finally, after increasing the temperature at 0.5 °C/min, 94 °C for 15 s. Quantitation was performed using StepOne software ver. 2.3 (Applied Biosystems, USA), employing perilla *β-actin* as a reference gene. Primers were designed with the GenScript website for real-time PCR primer design to generate approximately 200 bp amplicons with a 55 °C melting temperature (https://www.genscript.com/ssl-bin/app/primer). Primers for qRT-PCR are listed in Additional file 1: Table S1.

## Results

### *PcD6DES* gene cloning and sequence analysis

**Fig. 1:**
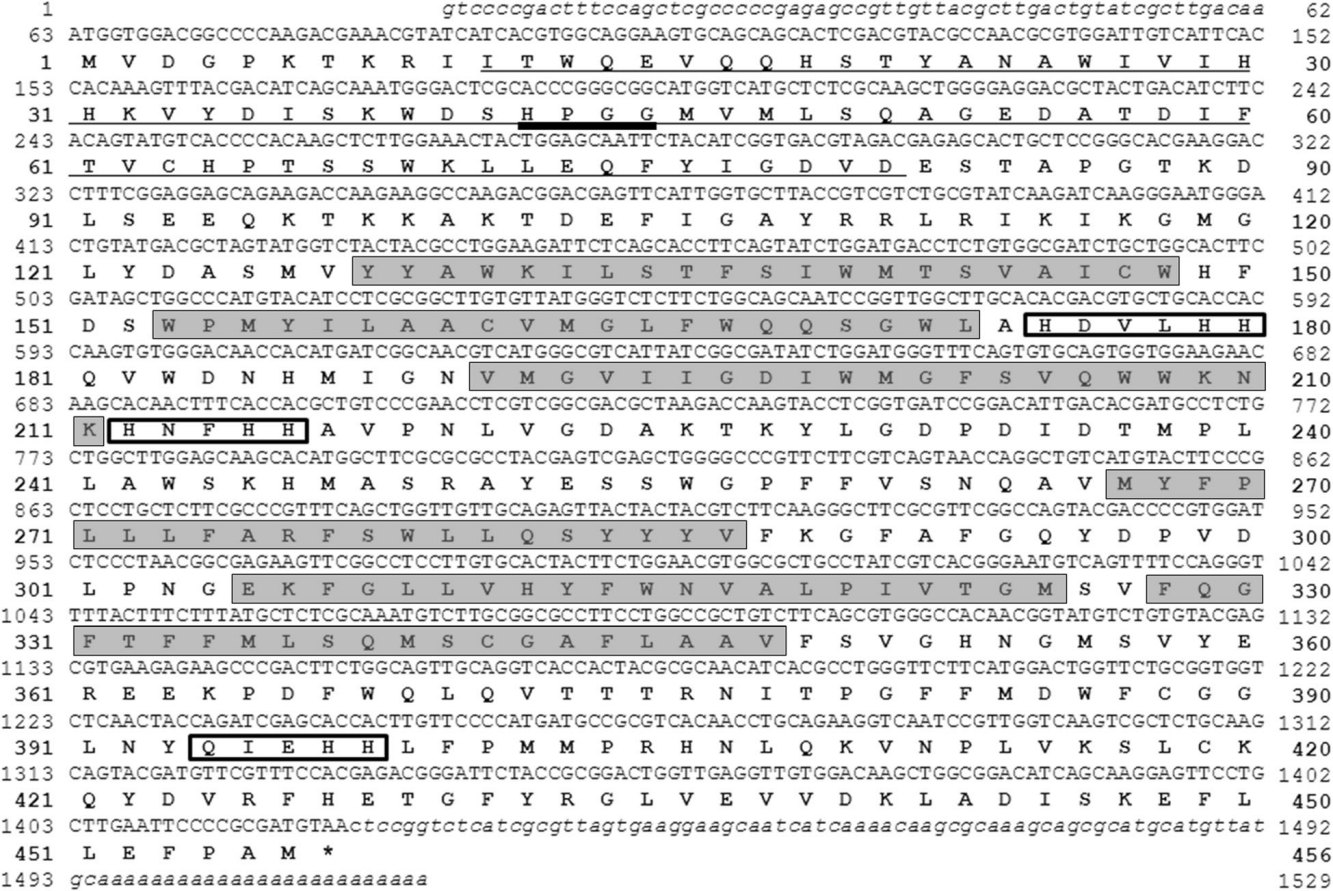
Nucleotide and deduced amino acid sequences of the *PcD6DES* gene. Lowercase italics and capital letters indicate 5′/3′-untranslated region and coding sequence, respectively. Bold capital letters represent amino acid residues. The cytochrome *b5* domain (the main characteristic of a front-end desaturase) is underlined. Thick underline indicates the heme-binding motif, HPGG. Gray and white boxes indicate the transmembrane domain and histidine box, respectively. Asterisk indicates the translation termination sequence

We began by cloning the full-length *PcD6DES* mRNA, which was 1529 bp with a 1371 bp sequence encoding 456 aa. Residues 11 to 81 were predicted to form a cyb5 domain, which is characteristic of front-end desaturases (Fig. 1) [20]. In addition to the cyb5 domain, fatty acid desaturases contain the heme-binding motif HPGG-X_8_-G-X_6_-F-X_3–6_-H known as HPGG [19]. *PcD6DES* encodes a protein with an HPGG motif at 42–45 aa, with the sequence HPGG-X_7_-G-X_6_-F-X_3_-H, in the cyb5 domain. In addition, PcD6DES has three histidine boxes (His boxes): HDVLHH, HNFHH and QIEHH. His boxes are the most important characteristics of fatty acid desaturases, as they determine their desaturase function [29, 30]. The His boxes of common fatty acid desaturases have the sequences HXXXH, HXXHH and HXXHH. Importantly, the third His box of the front end desaturase has the sequence QXXHH, and it appears that the Q residue mediates desaturase function [30]. The sequences of the His boxes of PcD6DES (HDVLHH, HNFHH and QIEHH) are consistent with the conserved His box motifs HXXXH, HXXHH and QXXHH of D6DES (Fig. 1) [30]. Finally, most membrane-bound fatty acid desaturases have four to six transmembrane domains (TMs) [31–33], and PcD6DES was predicted to have six TM domains (Fig. 1, Additional file 2: Figure S2). TM1 to TM6 were predicted to be located sequentially at 128–148 aa, 153–173 aa, 191–211 aa, 267–287 aa, 305–325 aa and 328–348 aa (Fig. 1, Additional file 2: Figure S2).The TMs patterns of of PcD6DES, evening primrose D6DES, perilla FAD2 are very similar to among them (Additional file 2: Figure S2).

### Phylogenetic analysis

**Fig. 2:**
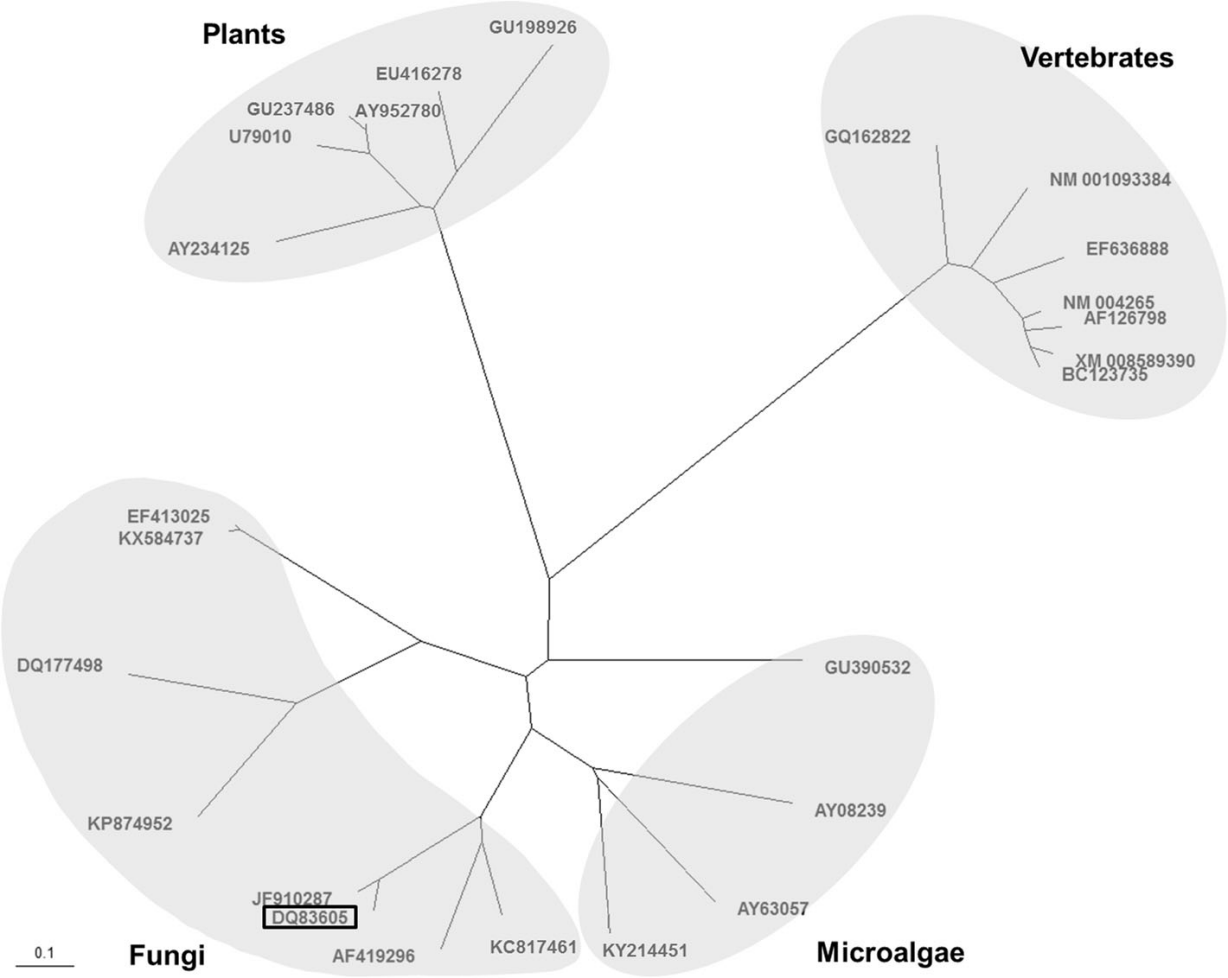
Phylogenetic analysis of the deduced amino acid sequences of *D6DES* genes from various organisms. D6DES from microalgae (Protista), fungi (Fungi), plants (Plantae) and vertebrates (Animalia) are separately grouped by kingdom. Letters and numbers represent GenBank accession numbers. The origin of each D6DES is as follows: AF126798, *Mus musculus*; AF419296, *Pythium irregulare*; AY08239, *Phaeodactylum tricornutum*; AY234125, *Primula farinosa*; AY63057, *Glossomastix chrysoplasta*; AY952780, *Echium plantagineum*; BC123735, *Bos taurus*; DQ177498, *Cunninghamella echinulata*; DQ83605, *Phytophthora citrophthora*; EF413025, *Mortierella alpina*; EF636888, *Gallus gallus*; EU416278, *Oenothera biennis*; GQ162822, *Sparus aurata*; GU198926, *Ribes nigrum*; GU237486, *Echium amoenum*; GU390532, *Parietochloris incisa*; JF910287, *Phytophthora infestans*; KC817461, *Pythium aphanidermatum*; KP874952, *Umbelopsis isabellina*; KX584737, *Mortierella alpina*; KY214451, *Nannochloropsis oceanica*; NM_001093384, *Xenopus laevis*; NM_004265, *Homo sapiens*; U79010, *Borago officinalis*; XM_008589390, *Galeopterus variegatus*. Open box indicates PcD6DES. Sequences were aligned using DNASTAR MegAlign (Ver. 8.1.4) with the ClustalW method. The phylogenetic tree was generated using TreeView (Ver. 1.6.6) with the aligned data. Scale bar indicates 0.1 amino acid substitution per site

We performed phylogenetic analysis of D6DES peptide sequences from 25 species belonging to the Protista, Fungi, Plantae and Animalia kingdoms (Fig. 2). The D6DES sequences formed groups within each kingdom. The identity between PcD6DES (DQ3605) and PinD6DES (JF910287), which were the first and second reported D6DES in the *Phytophthora* genus, respectively, was 90.8%. However, the identity between PcD6DES and other fungal D6DES proteins was at most 64.1%. Furthermore, for the Fungi and Protista, the identities among deduced amino acids of *D6DES* genes were low (38.2 and 55.2%, respectively).

### Functional analysis in *Saccharomyces cerevisiae*

We compared the fatty acid composition between the control yeast strain and pYES2-*PcD6DES* yeast to investigate the production of SDA and GLA and confirm PcD6DES function. Above all, the expression of *PcD6DES* in transformed yeast cells was confirmed using RT-PCR (Additional file 2: Figure S3). Yeast harboring the pYES2 empty vector, which served as a control, only produced hexadecanoic acid (palmitic acid; C16:0), cis-9-hexadecanoic acid (palmitoleic acid; C16:1Δ^9^), octadecanoic acid (stearic acid; C18:0) and cis-9-octadecenoic acid (oleic acid; C18:1Δ^9^). Furthermore, when provided with LA, this control yeast strain did not produce any other fatty acids. However, pYES2-*PcD6DES* yeast supplemented with LA produced GLA. In addition, pYES2-*PcD6DES* yeast supplied with LA and ALA synthesized SDA as well as GLA (Table 1). These results demonstrate that the *PcD6DES* gene encodes a protein that can convert LA and ALA to GLA and SDA, respectively, in yeast.

**Table 1:** Fatty acid composition of pYES2-*PcD6DES* yeast cultured in medium containing fatty acid substrates. Trace indicates below 0.1 mole%. Data represent mole% of fatty acid methyl esters. Experiments were carried out in triplicate, and the mean values are displayed

Fatty acid	pYES2		pYES2-*PcD6DES*	
	-	+LA	+LA	+LA and ALA
C16:0	17.4	18.1	20.7	15.9
C16:1Δ^9^	41.2	15.2	15.8	6.8
C16:2Δ^6,9^	-	-	3.1	0.6
C18:0	7.2	7.1	7.9	7.7
C18:1Δ^9^	34.2	13.8	15.8	8.3
C18:2Δ^6,9^	-	-	trace	trace
LA	-	45.8	25.1	21.5
GLA	-	-	11.6	5.3
ALA	-	-	-	25.1
SDA	-	-	-	8.8
Conversion rate (%)	-	-	26.4	21.6
Saturated FA: unsaturated FA	1:3.07	1:2.97	1:2.50	1:3.24

We also analyzed the conversion rate of products by PcD6DES. In the case of pYES2-*PcD6DES* supplied with LA, the conversion rate of GLA was 11.6/(25.1 + 11.6) = 31.6%, putative C16:2Δ^6,9^ was 3.1/(15.8 + 3.1) = 16.4% and the total conversion rate was 26.4% (Table 1). In the case of pYES2-*PcD6DES* supplied with both LA and ALA, the conversion rate of GLA was 5.3/(21.5 + 5.3) = 19.8%, SDA was 8.8/(25.1 + 8.8) = 26.0%, putative C16:2Δ^6,9^ was 0.6/(6.8 + 0.6) = 8.1% and the total conversion rate was 21.6% (Table 1). The ratios of saturated fatty acids to unsaturated fatty acids were not significantly different in yeast cultures supplemented with LA and/or ALA and without LA and/or ALA (Table 1). A putative cis-6,9-octadecadienoic acid (C16:2Δ^6,9^) peak was detected in the samples and putative C18:2Δ^6,9^ was also detected, but at a much lower proportion than C16:2Δ^6,9^ (Table 1).

### Fatty acid analysis and segregation ratio of transgenic perilla

**Fig. 3:**
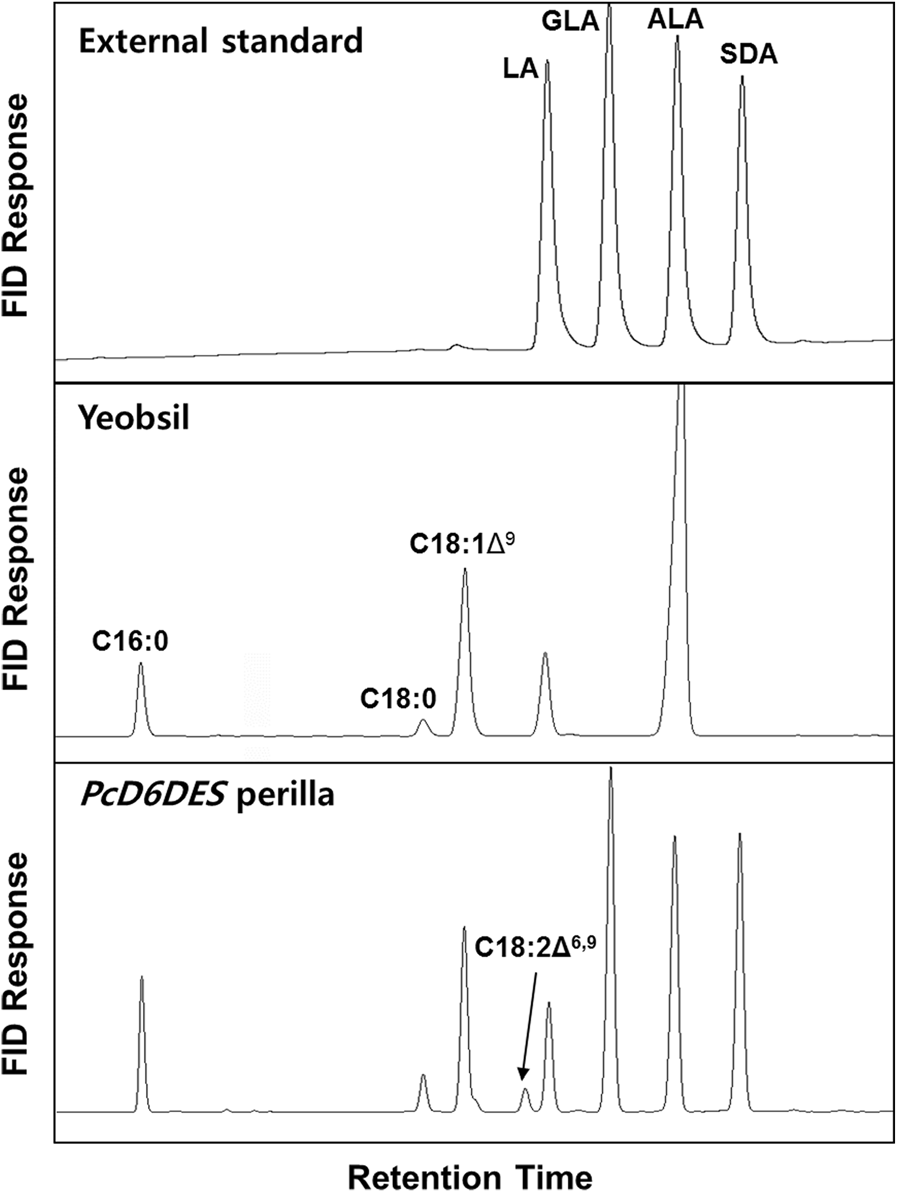
Chromatogram of *PcD6DES* perilla analyzed by GC. External standard for the mixture of LA, GLA, ALA and SDA was transmethylated. Yeobsil perilla seeds contain common fatty acids C16:0, C18:0, C18:1Δ^9^, LA and ALA. *PcD6D* transgenic perilla seeds contain C18:2Δ^6,9^, GLA and SDA, which are absent in Yeobsil

Five T_0_ *PcD6DES* transgenic perilla plants (PD6Ds) were obtained by *Agrobacterium*-mediated transformation. The transformants were confirmed by PCR of the *PcD6DES* gene and phosphinothricin acetyltransferase gene, which confers resistance to the herbicide Basta (Bayer Crop Science, Republic of Korea). We analyzed the fatty acid compositions of the T_2_ PD6D seeds from two T_1_ PD6Ds of each line (Table 2). Three new peaks, which were absent in Yeobsil, appeared in the chromatogram from PD6D seeds (Fig. 3). The retention times of two peaks of them, located earlier and later than that of ALA, are the same as that of GLA and SDA in the external standard, respectively (Fig. 3). The remaining new peak that appeared in front of the LA peak was putative C18:2Δ^6,9^, as expected (Fig. 3), as this compound is found in some fungi (Table 2) [12, 13]. In transgenic PD6D plants, the proportions of GLA, SDA and putative C18:2Δ^6,9^ increased while the proportions of oleic acid, LA and ALA decreased. In particular, the ALA content was significantly reduced in PD6D, likely due to GLA and SDA synthesis expending their respective substrates LA and ALA. Oleic acid content decreased in PD6D compared with Yeobsil (Table 2), likely as a consequence of increasing PUFA content. There were two types of T_1_ PD6Ds whose T_2_ seeds contained over 46% or 28–35% GLA and SDA content in seed oil, respectively. We hypothesized that the variance resulted from the difference in *PcD6DES* gene expression levels between the homozygote and hemizygote. To measure the segregation ratio, we treated 40–50 T_1_ seedlings with 0.3% Basta and investigated the segregation ratios of the progenies of four T_0_ PD6D lines. All four T_1_ perilla lines segregated at a ratio of 3:1 (Additional file 1: Table S2).

**Table 2:** Fatty acid composition of mature *D6DES* T_2_ perilla (PD6D) seeds. Data represent mole% of fatty acid methyl esters. Experiments were performed in triplicate, and the mean values are given

Fatty acid	Yeobsil	PD6D 1-1	PD6D 1-3	PD6D 2-1	PD6D 2-3	PD6D 3-1	PD6D 3-3	PD6D 4-1	PD6D 4-3
C16:0	7.7	7.3	7.1	7.2	7.2	7.3	7.6	7.5	7.3
C18:0	2.4	2.6	2.6	2.3	2.4	2.7	2.7	2.4	2.2
C18:1Δ^9^	18.5	15.7	14.4	12.9	14.7	14.2	13.3	13.7	12.6
C18:2Δ^6,9^	-	1.6	2.0	0.7	1.8	1.1	1.7	1.8	0.6
LA	11.5	9.3	8.1	9.8	8.6	10.4	7.7	7.2	11.6
GLA	-	16.7	24.9	15.9	24.6	16.8	24.8	24.4	14.2
ALA	60.1	29.9	20.8	32.8	21.0	30.2	21.0	21.5	37.6
SDA	-	16.9	20.2	18.5	19.7	17.4	21.1	21.5	13.8
D6DES products	-	35.1	47.0	35.1	46.1	35.3	47.6	47.7	28.7

### Confirmation of the putative C18:2Δ^6,9^ peak

**Fig. 4:**
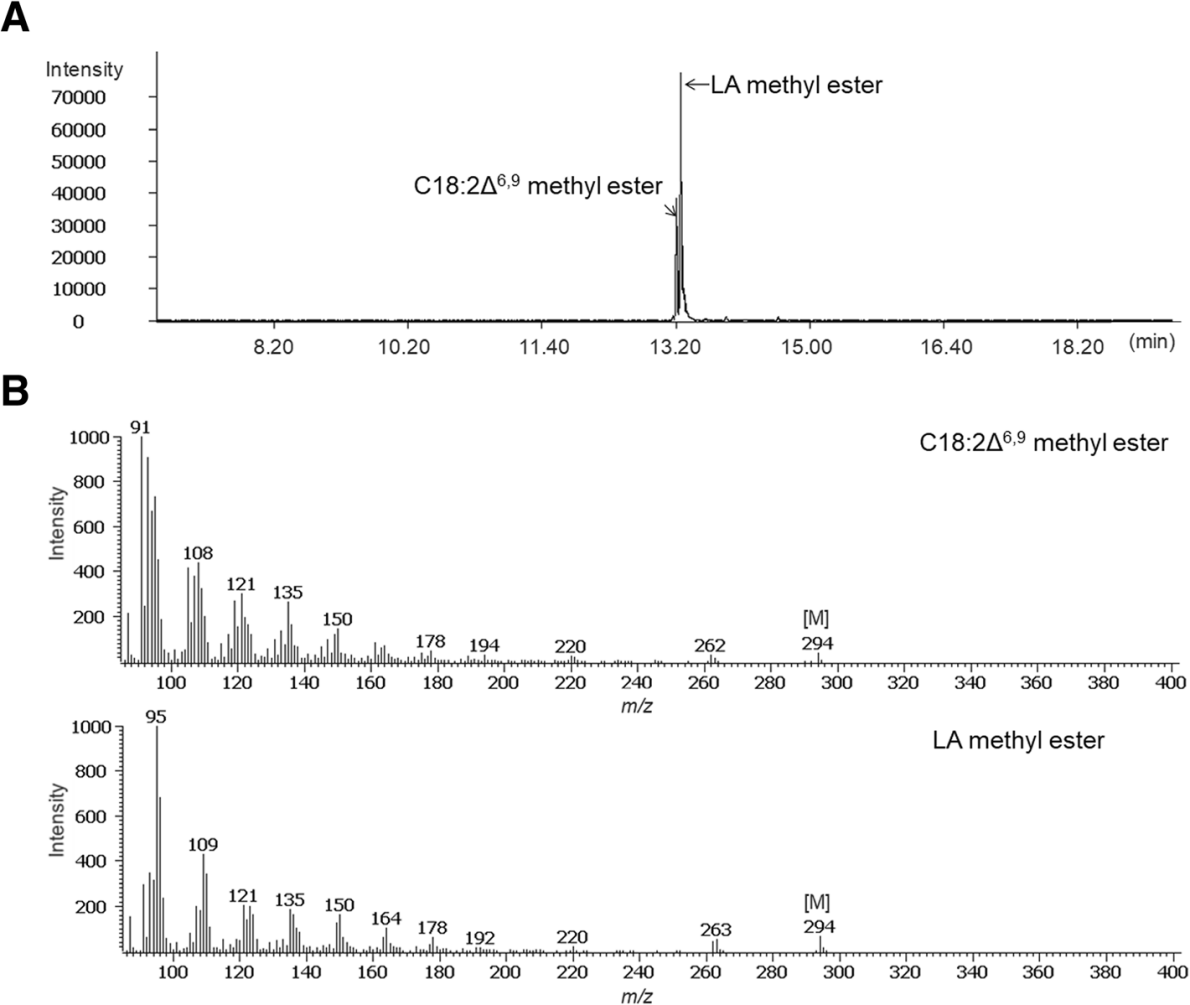
Selected-ion chromatogram and mass spectra for LA methyl ester and putative C18:2Δ^6,9^ methyl ester (molecular mass: 294) from *PcD6DES* perilla seed. **a** Selected-ion chromatogram for m/z 294 and (**b**) mass spectra of LA methyl ester and putative C18:2Δ^6,9^ methyl ester as methyl ester derivatives separated on a 30 m*0.25 mm i.d. fused-silica capillary column coated with 0.25 μm CP-SIL 8 CB Low Bleed

To confirm the identity of the putative C18:2Δ^6,9^, we analyzed this compound from *PcD6DES* perilla seed oil using Pegasus HT GC-TOF MS (LECO, USA). LA and C18:2Δ^6,9^ are isomers whose double bond positions differ from each other. In the GC-TOF MS results, two slightly different peaks and mass spectra were observed (Fig. 4). In addition, these peaks were identified by matching their mass spectra from the NIST11 and Wiley9 mass libraries. Finally, the peak between the oleic acid and LA peaks was possibly C18:2Δ^6,9^ .

### *PcD6DES* expression in leaves and developing seeds of transgenic *PcD6DES* perilla

**Fig. 5:**
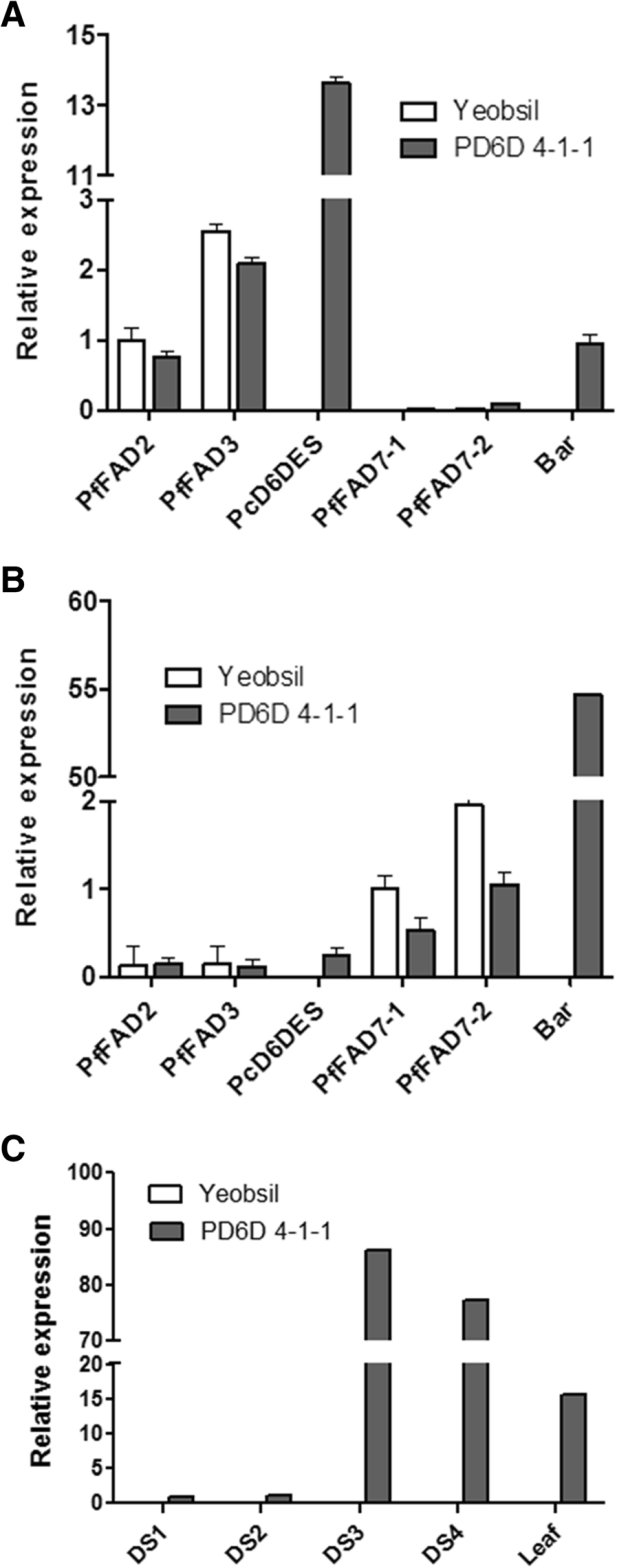
Relative expression levels of *PfFAD2*, *PfFAD3*, *PfFAD7–1*, *PfFAD7–2*, *PcD6DES*, and *bar* in (**a**) DS3, (**b**) leaves and (**c**) that of *PcD6DES* in DS1 to DS4 and leaf using quantitative RT-PCR. All experiments were carried out three times, and error bars indicate standard deviation

We performed quantitative RT-PCR (qRT-PCR) to measure the expression levels of *PcD6DES* and other fatty acid desaturase genes in leaves and developing seeds from Yeobsil and PD6D (Fig. 5). In DS3 of Yeobsil or PD6D 4–1-1, the expression of *fatty acid desaturase 2* gene from *P. frutescens* (*PfFAD2*) and *PfFAD3* was relatively high compared to that of *PfFAD7–1* and *PfFAD7–2*. *PcD6DES* was highly expressed during DS3 of PD6D 4–1-1; however, it was not expressed in Yeobsil at DS3 (Fig. 5a). In leaves of Yeobsil or PD6D 4–1-1, *PfFAD7–1* and *PfFAD7–2* were expressed at higher levels than *PfFAD2* and *PfFAD3*. *PcD6DES* was expressed at low levels similar to those of *PfFAD2* and *PfFAD3* (Fig. 5b). *Bar* (Basta resistance) was expressed during DS3 and in leaves of PD6D 4–1-1, but not in either DS3 or leaves of Yeobsil (Fig. 5a, b). In particular, the expression level of *bar* was much higher (54.7-fold) than that of other genes in PD6D 4–1-1 leaves (Fig. 5b). We also determined the expression level of *PcD6DES* gene in DS1 to DS4 and leaf. The expression of *PcD6DES* gene in PD6D 4–1-1 was low in DS1 to DS2 and very high in DS3 to DS4 (Fig. 5c). *PcD6DES* gene was also highly expressed in leaf tissue more than expected. No expression was detected in all Yeobsil samples.

### Fatty acid composition in *PcD6DES* transgenic perilla leaves

To investigate the changes in fatty acid composition in PD6D leaves, we analyzed the fatty acid composition of 4-week-old leaves from T_2_ homozygous transgenic plants. Unlike the fatty acid composition of perilla seeds, leaves contained much higher levels of C16 fatty acids and LA but lower levels of oleic acid and ALA (Table 3). Although the expression of *PcD6DES* was driven by the seed-specific vicilin promoter from *Pisum sativum* [34], GLA and SDA were still synthesized and accumulated in PD6D leaves, albeit at a lower proportion than in seeds (Table 3). PD6D seeds showed similar proportions of fatty acids among PD6D homozygous lines (PD6D #1–3, #2–3, #3–3, #4–1) (Table 2). However, PD6D leaves exhibited different proportions of the fatty acids LA, GLA and ALA among PD6D homozygous lines, and C18:2Δ^6,9^ was not detected in leaves (Table 3).

**Table 3:** Fatty acid composition of leaves from *D6DES* T_2_ perilla (PD6D) and Yeobsil plants. Data represent mole% of fatty acid methyl esters. Experiments were performed in triplicate, and the mean values are shown

Fatty acids	Yeobsil	PD6D 1-3-1	PD6D 2-3-2	PD6D 3-3-2	PD6D 4-1-1
C16:0	25.5	21.5	22.2	24.7	20.4
C16:1Δ^3t^	3.3	3.2	3.2	3.1	2.8
C16:2	0.9	0.5	0.6	0.6	0.4
C16:3	2.5	3.1	3.4	3.7	3.3
C18:0	3.3	4.5	4.3	4.0	4.4
C18:1Δ^9^	4.1	3.5	3.6	4.1	3.3
LA	23.0	14.5	13.9	11.3	9.5
GLA	-	6.0	8.8	10.1	6.7
ALA	35.7	37.3	33.1	31.9	42.9
SDA	-	4.2	5.1	5.1	4.9
C20:0	1.8	1.9	1.8	1.6	1.5
*D6DES* products	0.0	10.1	13.9	15.2	11.6
Total PUFA	62.1	65.5	64.8	62.5	67.7

### Fatty acid composition in developing seeds from *PcD6DES* transgenic perilla

GLA and SDA accumulated to high levels in PD6D mature seeds. We measured GLA and SDA, as well as fatty acids, at different stages of seed development to determine how the compositions of these molecules changed throughout seed development (Table 4). During early seed development in Yeobsil, saturated fatty acid content was relatively high compared to the late stage. In addition, the proportions of LA and GLA were similar. However, as the seeds approached the late stage of development, saturated fatty acid contents became lower and a large proportion of LA was converted to ALA. The fatty acid composition in developing seed stage 1 (DS1) and the mature stage DS4 resembled that of mature perilla leaves. The saturated fatty acid content in PD6D DS1 was almost the same as that in Yeobsil DS1. During late seed development, saturated fatty acid contents were lower and PUFAs contents were higher than at the early stage in both Yeobsil and PD6D.

**Table 4:** Fatty acid composition of developing seeds from *PcD6DES* T_2_ perilla (PD6D) and Yeobsil plants. Data represent mole% of fatty acid methyl esters. Experiments were repeated in triplicate, and the mean values are given

Fatty acid	Yeobsil				PD6D 4-1-1			
	DS1	DS2	DS3	DS4	DS1	DS2	DS3	DS4
C16:0	21.9	20.4	12.1	8.2	21.8	19.3	11.9	8.0
C18:0	6.2	6.5	2.5	2.1	6.3	6.9	2.6	2.0
C18:1Δ^9^	6.0	5.9	10.1	10.7	5.0	5.4	8.8	11.9
C18:2Δ^6.9^	-	-	-	-	-	-	0.3	0.9
LA	32.3	30.4	19.5	18.6	32.7	28.9	14.4	7.8
GLA	-	-	-	-	2.2	3.1	18.0	25.4
ALA	33.7	36.9	55.8	60.4	30.3	32.0	25.3	22.0
SDA	-	-	-	-	1.7	4.4	18.7	22.0

**Fig. 6:**
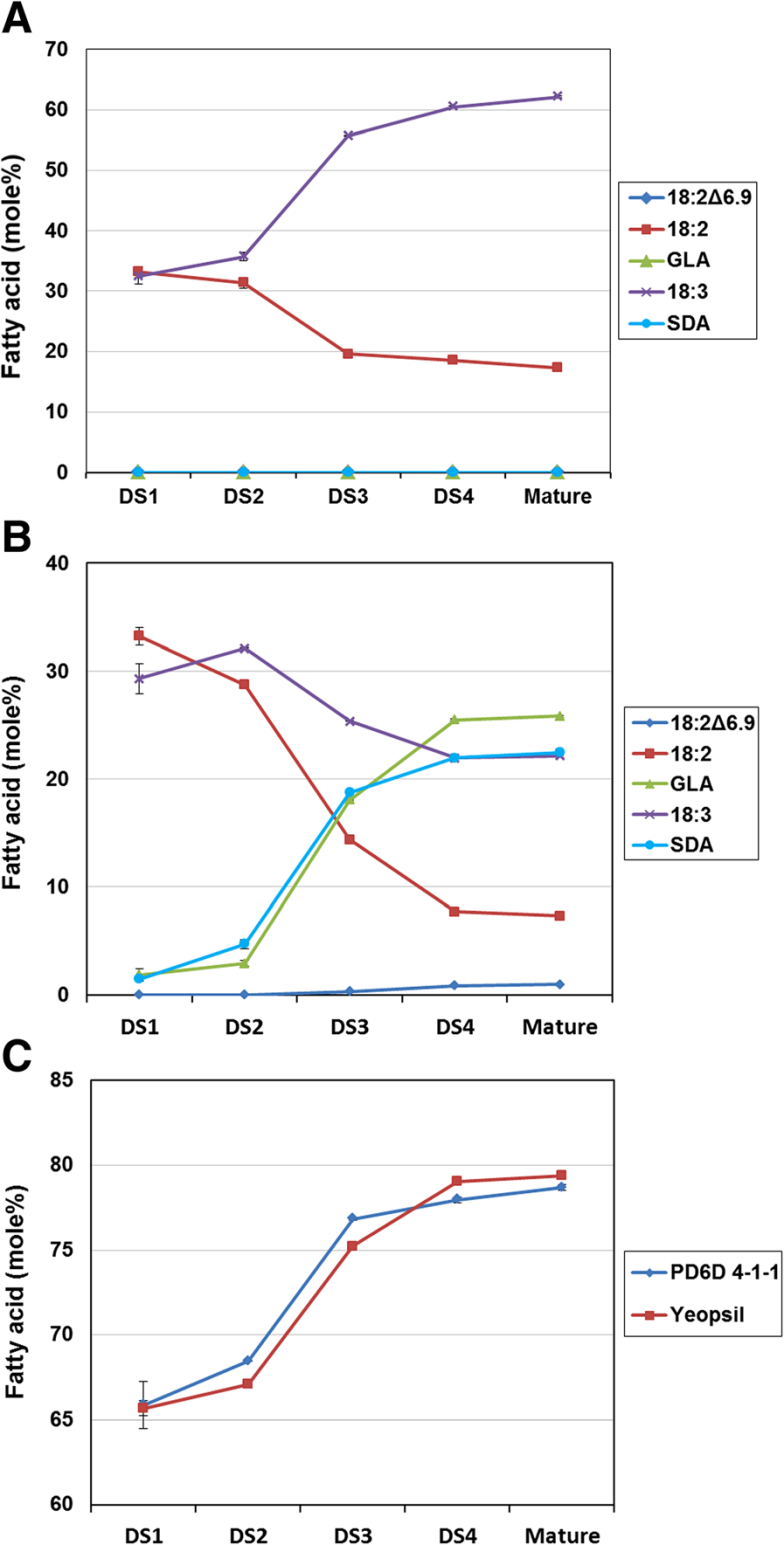
Changes in the proportion of each PUFA in (**a**) Yeobsil, (**b**) PD6D, and (**c**) changes in the proportion of total PUFAs during seed development

The change in the proportions of PUFA during seed development in Yeobsil and PD6D are shown in Table 4 and Fig. 6. In Yeobsil, the proportions of LA and ALA were similar in DS1, and after DS1, LA levels gradually decreased, whereas ALA levels gradually increased (Table 4, Fig. 6a). In PD6D seeds, the proportions of fatty acids did not differ from those of Yeobsil in DS1. However, after DS1, the proportion of ALA decreased and SDA increased at the expense of ALA. In addition, the proportion of LA was lower than that of Yeobsil, and GLA appeared to be produced from LA (Table 4, Fig. 6b). PUFAs were diversified, and the degree of unsaturation became higher in PD6D seeds, which is similar to Yeobsil at all stages of seed development (Fig. 6c).

### Fatty acid analysis of neutral lipids and polar lipids in mature perilla seeds

To investigate the differences in fatty acid compositions of polar lipids and neutral lipids from mature Yeobsil and PD6D (DS4) seeds, we extracted total lipids from the seeds, separated them using TLC (Additional file 2: Figure S4) and analyzed the fatty acid composition of each lipid using GC. The fatty acid compositions of neutral lipids, TAG, diacylglycerol and polar lipids were analyzed separately. The fatty acid composition of polar lipids in mature Yeobsil DS4 seeds was similar to that at DS1 (Tables 4 and 5). The polar lipids from mature PD6D seeds contained more GLA and SDA than those of PD6D DS1 (Table 5). GLA and SDA associated more with neutral lipids than with polar lipids in PD6D but were not detected in Yeobsil seeds.

**Table 5:** Fatty acid composition of neutral lipids and polar lipids from mature perilla seeds. Data represent mole% of fatty acid methyl esters. Experiments were repeated in triplicate, and the mean values are presented

	Polar lipids		Neutral lipids	
	Yeobsil	PD6D 4-1-1	Yeobsil	PD6D 4-1-1
C16:0	20.2	22.2	7.8	7.4
C18:0	7.2	7.9	2.3	2.3
C18:1Δ^9^	6.8	9.1	11.6	13.3
C18:2Δ^6.9^	-	-	-	1.3
LA	30.1	19.1	17.6	7.2
GLA	-	15.7	-	25.9
ALA	35.7	19.8	60.8	21.8
SDA	-	6.2	-	20.8

### Seed weight and seed oil content of transgenic *PcD6DES* perilla

**Fig. 7:**
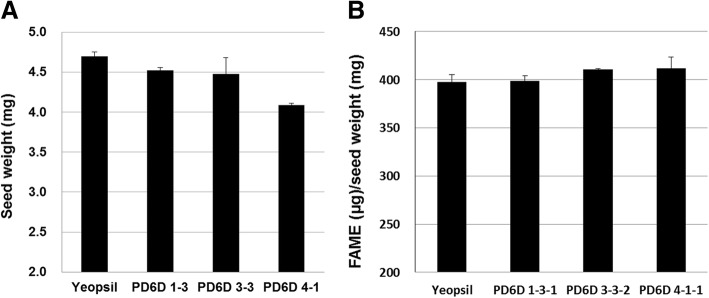
Analysis of (**a**) weight and (**b**) oil content of 100 WT perilla and *PcD6DES* perilla seeds. All experiments were performed in triplicate, and error bars indicate standard deviation

To investigate the characteristics of transgenic *PcD6DES* perilla seeds compared with Yeobsil seeds, we measured seed weight and oil content. We weighed 100 seeds three times and calculated the average seed weights. The seed weight of Yeobsil was the highest (4.70 mg/seed), and average seed weight of T_2_ perilla seeds was slightly less (4.36 mg/seed; 4.09–4.52 mg/seed) than Yeobsil seeds (Fig. 7a). The seed weights measured in this study are similar to those reported by Asif [22]. Meanwhile, the seed oil content showed different trends compared to seed weight. The fatty acid contents in PD6D seeds were 397.6 μg/mg seeds and was similar or 3.3–3.6% higher than that of Yeobsil seeds. The fatty acid contents in PD6D 1–3-1, 3–3-2 and 4–1-1 were 398.9, 410.6 and 411.8 μg/mg seeds, respectively (Fig. 7b), and the average seed oil content was 407.1 μg/mg seeds. Based on these data, the FAME levels per seed were as follows: Yeobsil, 1.87 mg FAME/seed; PD6D 1–3-1, 1.80 mg FAME/seed; PD6D 3–3-2, 1.84 mg FAME/seed; PD6D 4–4-1, 1.68 mg FAME/seed. The FAME levels per seed in the PD6D lines were almost the same or 89.8% those of Yeobsil seeds. The total seed oil content was approximately 40% in perilla, which is in agreement with the data from Asif [22] and Shin and Kim [24]. Compared to Yeobsil, the seed weight of PD6D was slightly lower, but these seeds possessed slightly higher oil contents.

## Discussion

In this study, we characterized PcD6DES, the first D6DES identified in *Phytophthora* spp., and found it to resemble other known fatty acid desaturases. PcD6DES contains cytochrome b5 domain, three histidine boxes and six TM domains (Fig. 1). Thus, we conclude that *PcD6DES* encodes a protein with characteristics of a ‘front-end’ fatty acid desaturase.

*Saccharomyces cerevisiae* has only one fatty acid desaturase, Ole1p which catalyzes the conversion C16:0 and C18:0 into C16:1Δ^9^ and C18:1Δ^9^, respectively [35]. Therefore, *S. cerevisiae* contains mainly four kinds of fatty acids, C16:0, C16:1Δ^9^, C18:0 and C18:1Δ^9^. Functional characterization of *PcD6DES* in budding yeast showed PcD6DES produce C16:2Δ^6,9^ and C18:2Δ^6,9^, although the proportion of C18:2Δ^6,9^ was barely detectable (Table 1). This result suggests that PcD6DES might have a weak affinity for monounsaturated fatty acids, including C16:1Δ^9^ and C18:1Δ^9^ (Table 1). The ratios of saturated fatty acids to unsaturated fatty acids did not differ much from those of yeast cultured with LA and/or ALA and without LA and/or ALA. These results likely indicate that fatty acid metabolism is regulated in yeast cells to maintain the appropriate levels of unsaturated fatty acids while they take up unsaturated fatty acids. However, unlike the fatty acid composition of budding yeast expressing *PcD6DES*, we detected C18:2Δ^6,9^ in *PcD6DES* perilla seeds (Fig. 3). This is likely because perilla seed oil contains little C16:1Δ^9^ and relatively high levels of C18:1Δ^9^, which is consistent with reports describing the detection of C18:2Δ^6,9^ in transgenic oilseed crops expressing a fungal *D6DES* [12, 13].

The sum of GLA and SDA levels from each progeny of the same T_0_ plant was highly variable (Table 2, Additional file 1: Table S3). We divided the progenies into those with high and low contents of D6DES product, which we attributed to the presence of the transgene in a homozygous vs. hemizygous state. The results of genotyping by Basta treatment supported this hypothesis (Additional file 1: Table S3). Under Basta treatment, seedlings of the high GLA and SDA lines (putative homozygous lines) all survived, whereas those of low GLA and SDA lines (putative hemizygous lines) showed a 3:1 segregation ratio (Additional file 1: Table S3). Therefore, it appears GLA and SDA contents depend on zygosity, with higher levels seen in homozygous plants and lower levels detected in hemizygous plants.

*PcD6DES* was expressed and PcD6DES products accumulated not only in seeds but also in leaves of transgenic perilla (Table 3; Fig. 5), despite *PcD6DES* being expressed from a seed-specific *vicilin* promoter. It is hard to conclude vicilin promoter shows leaky expression pattern because the expression pattern of vicilin promoter from *Pisum sativum* has not been reported. We supposed a few possibilities which results in this phenomenon. First, vicilin promoter from *Pisum sativum* might be originally leaky promoter which is not regulated tightly. Second, when vicilin promoter moved to perilla from pea, it may not be spatially regulated in the same manner as it is in native species. There was a report when the seed-specific promoters from wheat and barley directed the expression of green fluorescence protein, the expression was leaky [36]. Third, when T-DNA harboring the expression cassette of *PcD6DES* under the control of vicilin promoter was integrated into perilla genome, the spatial regulation of *PcD6DES* expression might be changed by positional effect. Although the expression of the *vicilin* promoter does not appear to be entirely exclusive to seed tissue, the expression of *PcD6DES* was 47.6-fold higher in seeds than in leaves. Seeds contained much higher levels of substrates for PcD6DES (71.6%) than leaves (58.7%) (Tables 2 and 3). This difference is likely due to the varied *PcD6DES* expression levels and the differential availability of precursor contents between seeds and leaves. When the borage *D6DES* gene was expressed under the control of the cauliflower mosaic virus 35S promoter in tobacco (*Nicotiana tabacum*), the levels of substrates and products of D6DES were 74.2 and 22.8%, respectively, and the conversion rate was 31.5% [20]. Meanwhile, in the current study, the levels of substrates and products of D6DES in transgenic perilla leaves were 58.7 and 15.2%, respectively, and the conversion rate was 26.0% (Table 3). This difference is likely because the constitutive 35S promoter drove *D6DES* expression more strongly in leaves than the seed-specific promoter used in the current study, as well as the higher substrate content in tobacco leaves than in perilla leaves. Overall, these results support our findings described above.

In the fatty acids of perilla leaves, C16:1Δ^3t^ is produced by FAD4 in chloroplasts [37]. D6DES can convert C16:1Δ^9^ and C18:1Δ^9^ into C16:2Δ^6,9^ and C18:2Δ^6,9^, respectively, in budding yeast (Fig. 2). Therefore, C16:1Δ^3t^, not a substrate for D6DES, is not converted into C16:2Δ^6,9^.

The fatty acid compositions of DS1 and polar lipids in mature seeds in Yeobsil were similar (Tables 4 and 5). Seeds at the early stage of development contain little TAG [23], instead mainly containing polar lipids. On the contrary, PD6D did not reflect this trend. PcD6DES products are thought to accumulate in polar lipids of mature PD6D seeds during seed development. The fatty acid compositions from DS4 and neutral lipids of mature seeds in both PD6D and Yeobsil were similar (Tables 4 and 5), likely because almost all seed oil comprises neutral lipids. Given that polar lipids incorporate more 16:0 at *sn-1* position than TAG [38], polar lipids and DS1 contains more 16:0 than DS4 and neutral lipids (Tables 4 and 5). Severe alteration of 16:0 from DS1 to DS4 was not by D6DES but by the relative proportion change of polar lipids and TAG. SDA of DS4 was gradually increased in PD6D because TAG content of DS4 is higher than earlier stage. TAG contains higher PUFAs than polar lipids in perilla (Table 5). In Table 4, Yeobsil DS4 contains more PUFAs than Yeobsil DS1. Thus, PD6D DS4 contains more GLA and SDA than PD6D earlier stage. The fatty acid composition of polar lipids from mature seeds indicated that GLA and SDA associate with membrane lipids and could not be completely transferred to TAG (Table 5).

The levels and patterns of *PfFAD2*, *PfFAD3*, *PfFAD7–1* and *PfFAD7–2* expression coincided with the previous report [33]. The expression levels of endogenous genes, including *PfFAD2* and *PfFAD3* in DS3 and *PfFAD7–1* and *PfFAD7–2* in leaves, were lower in PD6D than in Yeobsil (Fig. 5a, b). Perhaps the high expression levels of *PcD6DES* and *bar* genes in DS3 and leaves, respectively, influence the endogenous genes responsible for the synthesis of PUFAs and lowered their expression. The expression level of *PcD6DES* gene in PD6D 4–1-1 is much higher in late stage than early stage (Fig. 5c). This result is in accordance with the expression of seed storage protein. The PcD6DES products of developing seeds in PD6D 4–1-1 were low in DS1 (3.9%) and DS2 (7.5%) but dramatically increased in DS3 (37.0%) and DS4 (48.3%) (Table 4). The fatty acid analysis of developing seeds in PD6D 4–1-1 is consistent with the expression level of developing seeds in PD6D 4–1-1.

The most common omega-3 and omega-6 fatty acids in vegetable oil are ALA and LA, respectively. Notably, these fatty acids are essential for human health. Vegetable oils from oilseed crops such as corn, sunflower, safflower, sesame, cottonseed and soybeans contain high levels of omega-6 fatty acid but little omega-3 fatty acid. If the ratio of omega-3 to omega-6 fatty acids decreases below the recommended 1:4 ratio due to the overconsumption of omega-6 fatty acid oil or cooking food in omega-6 fatty acid, there could be detrimental consequences to health [39]. Studies have shown that a high intake of omega-6 fatty acids increases blood viscosity, vasospasm and vasoconstriction and decreases bleeding time [39]. The ratio between omega-3 and omega-6 in PD6D seed oil is 1.35:1, which certainly exceeds the healthy recommended ratio. Moreover, PD6D is more beneficial to health because it can bypass the rate-limiting step from ALA to SDA, allowing SDA to be more efficiently converted to DHA [3].

In PD6D seed oil, GLA and SDA accumulated to levels of up to 24 and 21%, respectively (Table 2). By contrast, borage seeds contain 20–25% GLA, and evening primrose seeds contain 10% GLA, but they do not contain SDA [20]. Blackcurrant seeds contain 15.8% GLA and approximately 2% SDA [40, 41], and hemp seeds contain 3.6% GLA and 2–3% SDA [10, 42]. Echium seeds contain 11.8% GLA and 13% SDA, which is the highest SDA content in natural land plants [43]. While the sum of GLA and SDA from the plant seeds described above is up to 25%, PD6D seeds contain a similar amount of GLA to borage and a higher content of SDA than echium (Table 2).

The seed weight in perilla measured in the current study (~ 4 mg) was similar to that measured by Asif [22]. We detected a seed oil content of approximately 40% in perilla, which is also in agreement with previous results [22, 24]. Indeed, the introduction of fatty acid desaturase genes such as *D6DES* has not previously been shown to increase seed weight or seed oil content. Furthermore, transgenic plants generally demonstrate poor agronomic performance in traits other than their modified target traits. In *PcD6DES* perilla with the beneficial transgene activity the overall plant phenotype was similar to that of Yeobsil.

There have been several reports of transgenic plants accumulating GLA and/or SDA in their seeds via the use of a *D6DES* transgene alone or in tandem with other fatty acid desaturase genes. In an early study, the expression of *D6DES* from cyanobacteria in transgenic tobacco resulted in GLA and SDA accumulation in seed oil [44]. This was the first report of the biotechnological production of GLA and SDA in transgenic plants. Later, transgenic tobacco constitutively expressing *D6DES* from borage (*Borago officinalis*) was found to accumulate 13% GLA and 10% SDA in its seeds [20]. In addition, canola plants coexpressing *D6DES* from the oleaginous fungus *Mortierella alpina* and *Δ12 desaturase* gene (*Brassica napus*) specifically in their seeds produced 40% GLA [12]. Similarly, when *D6DES* from the oleaginous fungus *Pythium irregulare* was expressed under the control of the seed-specific napin promoter in *Brassica juncea*, GLA comprised 40% of seed oil [13]. When the borage *D6DES* gene and Arabidopsis *Δ15 desaturase* gene were coexpressed in soybean driven by the soybean seed-specific *β-conglycinin* promoter, SDA accumulated to 29% of seed oil content [45]. Linseed expressing the SDA-specific *D6DES* gene from *Primula vialii* under the control of the *Vicia faba* seed-specific USP promoter had a 13% SDA content in its seed oil [15]. More recently, when the *D6DES* gene from the protozoan *Saprolegnia diclina* was expressed under the control of the Arabidopsis *OLEOSIN* promoter in safflower, GLA accumulated to 77% of the seed oil content, i.e., the highest reported GLA content for transgenic safflower [46]. It is important to note that the GLA levels in transgenic seed oil reported by Liu et al. [12], Hong et al. [13] and Nykiforuk et al. [46] were higher than those found in the current study. Furthermore, the SDA content in transgenic seed oil reported by Eckert et al. [45] was also higher than we achieved. However, the results of Liu et al. [12] and Eckert et al. [45] were achieved via coexpression of *D6DES* and another desaturase gene, and there has been no report of higher GLA and SDA production in transgenic seed oil due to the introduction of a single gene. In addition, the production of perilla seeds containing 47.7% D6DES products (GLA, SDA and C18:2Δ^6,9^) due to the incorporation of only *D6DES* gene represents a dramatic improvement (Table 2).

Looking forward, SDA could be produced at very high levels if the ALA-preferred *D6DES* were introduced into perilla. For example, D6DES enzymes from *Primula vialii* and *Primula luteola* showed high ALA substrate specificity and produced SDA exclusively [14, 47]. Furthermore, perilla that produces very high levels of SDA could be used to produce EPA and DHA by introducing *fatty acid elongase*, *Δ5 desaturase* and *Δ4 desaturase*.

## Conclusions

Two decades have been spent creating transgenic plants that produce higher levels of expensive functional fatty acids such as GLA and SDA in their seeds beyond what wild plants are capable of producing. In this study, we developed transgenic perilla with the very high content over 45% of both GLA and SDA. These plants might serve as an important resource for producing omega-3 oil capsules as health food and promoting human health. In addition, other genes could be added to these plants to create transgenic perilla that produce fish-oil-like oil in their seeds and provide further health benefits.

## Additional files

Additional file 1: **Table S1.** Primers used in this study. Nucleotide symbols are as follows: Y, C/T; R, G/A; W, A/T; D, G/A/T; N, A/T/G/C. **Table S2.** Segregation ratio of *D6DES* T_1_ perilla plants treated with Basta. **Table S3.** Genotyping of *D6DES* T_2_ perilla plants treatment with Basta. (ZIP 3970 kb)

Additional file 2: **Figure S1.** Vector constructs containing the PcD6DES gene. (A) Vector for yeast transformation. PGAL1 and CYS TT represent galactose-inducible GAL1 promoter and CYC1 transcriptional terminator, respectively. URA encodes a biosynthetic enzyme of uracil, as a marker gene for yeast selection. Amp^R^ encodes β-lactamase that inactivates antibiotics ampicillin, as a marker gene for *E. coli* selection. (B) Vector for plant transformation. pCAMBIA3300 was used as a backbone vector. Pvic and Tocs indicate vicilin promoter and octopine synthase III terminator, respectively. LB and RB represent left border and right border, respectively. Each box represents a gene expression cassette. B, *Bam*HI; C, *Cla*I; H3, *Hin*dIII; K, *Kpn*I; N, *Not*I; P, *Pst*I; R1, *Eco*RI; Sc, *Sac*I; X, *Xba*I, Xh, *Xho*I. **Figure S2.** The predicted transmembrane domains of fatty acid desaturases including **(A)** *Phytophthora citrophthora* D6DES, **(B)** evening primrose (*Oenothera biennis*) D6DES (GenBank accession No. EU416278) and **(C)** *Perilla frutescens var. frutescens* FAD2 (GenBank accession No. KP070823) by TOPCONS. **Figure S3.** The expression from *PcD6DES* gene in RNA level from *S. cerevisiae*. RT-PCR from total RNAs of *PcD6DES* yeast. pYES2 is yeast cells harboring a blank vector as an negative control. PcD6DES is yeast cells carrying pYES2-*PcD6DES*. *ScAct1* is a reference gene from *S. cerevisiae actin* gene (GenBank accession No. L00026). – and + indicate non-induction and induction, respectively. The induction method of yeast was described in Methods section. M, 1 kb DNA ladder. **Figure S4.** TLC analysis of lipids extracted from perilla mature seeds. The lipids were developed and visualized under the ultra violet after the primuline spraying. The spots corresponding neutral lipids (TAG and DAG) and polar lipids were scraped off and the fatty acid composition was analyzed with GC. The method using this experiment is described in the ‘Thin layer chromatography (TLC)’ subsection of Methods section (ZIP 537 kb)

## Abbreviations

ALA: α-linolenic acid
D6DES: Δ6 desaturase
DHA: cis-4,7,10,13,16,19-docosahexaenoic acid
DS: developing seed stage
EPA: cis-5,8,11,14,17-eicosapentaenoic acid
FID: flame ionization detector
GLA: γ-linolenic acid
LA: linoleic acid
SDA: stearidonic acid
TM: transmembrane domain
VLCPUFA: very long chain polyunsaturated fatty acid
