# Supplementary material for: High accumulation of γ-linolenic acid and Stearidonic acid in transgenic Perilla (Perilla frutescens var. frutescens) seeds
Source: BMC Plant Biol. 2019 Apr 1;19:120. doi: 10.1186/s12870-019-1713-2 (PMC6444538; doi:10.1186/s12870-019-1713-2)
Supplement: Supplementary file 1 — Table S1. Primers used in this study. Nucleotide symbols are as follows: Y, C/T; R, G/A; W, A/T; D, G/A/T; N, A/T/G/C. Table S2. Segregation ratio of D6DES T1 perilla plants treated with Basta. Table S3. Genotyping of D6DES T2 perilla plants treatment with Basta. (ZIP 3970 kb) [file 12870_2019_1713_MOESM1_ESM.zip › Supplementary Table 2.docx]

Supplementary Table 2. Segregation ratio of *D6DES* T_1_ perilla plants treated with Basta.

| Transgenic lines | Germination (A) | Survival (B) | Segregation ratio  B:(A-B) | χ^2^ statistical test^*^ | *p* value | Expected  copy number |
| --- | --- | --- | --- | --- | --- | --- |
| PD6D #1  PD6D #2  PD6D #3  PD6D #4 | 31  20  22  28 | 23  17  16  22 | 2.875:1  5.667:1  2.667:1  3.667:1 | 0.011  1.067  0.061  0.191 | 0.9172  0.3017  0.8056  0.6621 | 1  1  1  1 |

* When the value is lower than χ^2^_0.05_=3.841 (degrees of freedom=1), the result fits a 3:1 segregation ratio.
