# Supplementary material for: High accumulation of γ-linolenic acid and Stearidonic acid in transgenic Perilla (Perilla frutescens var. frutescens) seeds
Source: BMC Plant Biol. 2019 Apr 1;19:120. doi: 10.1186/s12870-019-1713-2 (PMC6444538; doi:10.1186/s12870-019-1713-2)
Supplement: Supplementary file 1 — Table S1. Primers used in this study. Nucleotide symbols are as follows: Y, C/T; R, G/A; W, A/T; D, G/A/T; N, A/T/G/C. Table S2. Segregation ratio of D6DES T1 perilla plants treated with Basta. Table S3. Genotyping of D6DES T2 perilla plants treatment with Basta. (ZIP 3970 kb) [file 12870_2019_1713_MOESM1_ESM.zip › Supplementary Table 3.docx]

Supplementary Table 3. Genotyping of *D6DES* T_2_ perilla plants treatment with Basta.

| Transgenic lines | GLA+SDA | Germination | Survival | Death | Resistant plants (%) | Genotype |
| --- | --- | --- | --- | --- | --- | --- |
| PD6D #1-1  PD6D #1-3  PD6D #2-1  PD6D #2-3  PD6D #3-1  PD6D #3-3  PD6D #4-1  PD6D #4-3 | 33.59  45.01  34.35  44.30  34.14  45.86  45.93  28.05 | 24  24  25  25  25  24  25  24 | 20  24  20  25  18  24  25  19 | 4  -  5  -  7  -  -  5 | 83.3  100.0  80.0  100.0  72.0  100.0  100.0  79.2 | Hemizygote  Homozygote  Hemizygote  Homozygote  Hemizygote  Homozygote  Homozygote  Hemizygote |
