# Supplementary material for: High accumulation of γ-linolenic acid and Stearidonic acid in transgenic Perilla (Perilla frutescens var. frutescens) seeds
Source: BMC Plant Biol. 2019 Apr 1;19:120. doi: 10.1186/s12870-019-1713-2 (PMC6444538; doi:10.1186/s12870-019-1713-2)
Supplement: Supplementary file 1 — Table S1. Primers used in this study. Nucleotide symbols are as follows: Y, C/T; R, G/A; W, A/T; D, G/A/T; N, A/T/G/C. Table S2. Segregation ratio of D6DES T1 perilla plants treated with Basta. Table S3. Genotyping of D6DES T2 perilla plants treatment with Basta. (ZIP 3970 kb) [file 12870_2019_1713_MOESM1_ESM.zip › Supplementary Table 1.docx]

Supplementary Table 1. Primers used in this study. Nucleotide symbols are as follows: Y, C/T; R, G/A; W, A/T; D, G/A/T; N, A/T/G/C.

| Primer name | Sequence (5’-3’) | Purpose |
| --- | --- | --- |
| D6N1  D6N2  D6C  5D6NP2  5D6P2  5D6NP1  5D6P1  5DN  5DC  ScAct F  ScAct R  FAD2 qRT F  FAD2 qRT R  FAD3 qRT F  FAD3 qRT R  FAD7-1 qRT F  FAD7-1 qRT R  FAD7-2 qRT F  FAD7-2 qRT R  PfAct qRT F  PfAct qRT R | TTYTGGCARCARTGYGGWTGGCTDGC  TGGTGGAARAAYAARCAYAAYAYNCAYTAY  GGTGGWYTGAAYTAYCARATHGARCAYCAY  CCGCAAGACATTTGAGAAGAGCAT  ACGTTCCAGAAGTAGTGCCACAA  GCTGGCTTGGAGCAAGCACATGGCTT  CGGCGACGCTAAGACCAAGTACCT  CGGGAAGCTTATTATGGTGGACGGCCCCAAG  CGATGGATCCGGAGTTACATCGCGGGGAAT  TATCGTCGGTAGACCAAGAC  ATTGGAACGACGTGAGTAAC  tcggcctagtcctccactcg  ggccttccggagacattgaa  tggccggtttactggattgc  Gcacccacgactcgtccttc  Tggccagtatctctccaattcca  ggaatggctgcccgaatatc  TTCAATTCTGGTGCCCTACC  CTTCCCGGGACTTCTACTCC  GGCAGTCCTCTCATTGTATGC  CTCGGCAGTTGTGGTAAACA | Degenerate PCR  Degenerate PCR  Degenerate PCR  5’ RACE  5’ RACE  3’ RACE  3’ RACE  Subcloning into vectors  Subcloning into vectors  RT-PCR of yeast RNA  RT-PCR of yeast RNA  Quantitative RT-PCR  Quantitative RT-PCR  Quantitative RT-PCR  Quantitative RT-PCR  Quantitative RT-PCR  Quantitative RT-PCR  Quantitative RT-PCR  Quantitative RT-PCR  Quantitative RT-PCR  Quantitative RT-PCR |
