# Supplementary material for: High accumulation of γ-linolenic acid and Stearidonic acid in transgenic Perilla (Perilla frutescens var. frutescens) seeds
Source: BMC Plant Biol. 2019 Apr 1;19:120. doi: 10.1186/s12870-019-1713-2 (PMC6444538; doi:10.1186/s12870-019-1713-2)
Supplement: Supplementary file 2 — Figure S1. Vector constructs containing the PcD6DES gene. (A) Vector for yeast transformation. PGAL1 and CYS TT represent galactose-inducible GAL1 promoter and CYC1 transcriptional terminator, respectively. URA encodes a biosynthetic enzyme of uracil, as a marker gene for yeast selection. AmpR encodes β-lactamase that inactivates antibiotics ampicillin, as a marker gene for E. coli selection. (B) Vector for plant transformation. pCAMBIA3300 was used as a backbone vector. Pvic and Tocs indicate vicilin promoter and octopine synthase III terminator, respectively. LB and RB represent left border and right border, respectively. Each box represents a gene expression cassette. B, BamHI; C, ClaI; H3, HindIII; K, KpnI; N, NotI; P, PstI; R1, EcoRI; Sc, SacI; X, XbaI, Xh, XhoI. Figure S2. The predicted transmembrane domains of fatty acid desaturases including (A) Phytophthora citrophthora D6DES, (B) evening primrose (Oenothera biennis) D6DES (GenBank accession No. EU416278) and (C) Perilla frutescens var. frutescens FAD2 (GenBank accession No. KP070823) by TOPCONS. Figure S3. The expression from PcD6DES gene in RNA level from S. cerevisiae. RT-PCR from total RNAs of PcD6DES yeast. pYES2 is yeast cells harboring a blank vector as an negative control. PcD6DES is yeast cells carrying pYES2-PcD6DES. ScAct1 is a reference gene from S. cerevisiae actin gene (GenBank accession No. L00026). – and + indicate non-induction and induction, respectively. The induction method of yeast was described in Methods section. M, 1 kb DNA ladder. Figure S4. TLC analysis of lipids extracted from perilla mature seeds. The lipids were developed and visualized under the ultra violet after the primuline spraying. The spots corresponding neutral lipids (TAG and DAG) and polar lipids were scraped off and the fatty acid composition was analyzed with GC. The method using this experiment is described in the ‘Thin layer chromatography (TLC)’ subsection of Methods section (ZIP 537 kb) [file 12870_2019_1713_MOESM2_ESM.zip › Supplementary Figure 3.pdf]

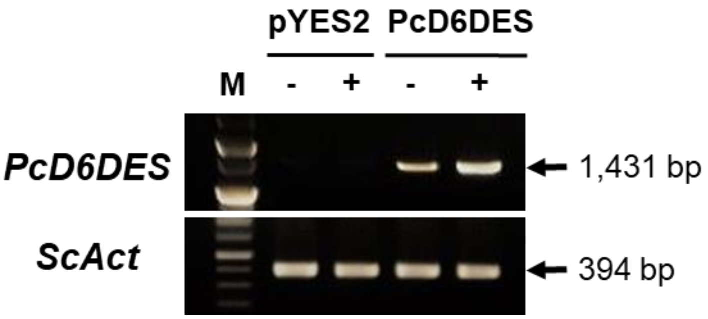

Fig. S3 The expression from *PcD6DES* gene in RNA level from *S. cerevisiae*. RT-PCR from total RNAs of *PcD6DES* yeast. pYES2 is yeast cells harboring a blank vector as an negative control. PcD6DES is yeast cells carrying pYES2-*PcD6DES*. *ScAct1* is a reference gene from *S. cerevisiae actin* gene (GenBank acceession No. L00026). – and + indicate non-induction and induction, respectively. The induction method of yeast was described in Methods section. M, 1 kb DNA ladder.
